# Gastrointestinal and Hepatotoxicity Assessment of an Anticancer Extract from Muricid Molluscs

**DOI:** 10.1155/2013/837370

**Published:** 2013-04-17

**Authors:** Chantel B. Westley, Kirsten Benkendorff, Cassandra M. McIver, Richard K. Le Leu, Catherine A. Abbott

**Affiliations:** ^1^School of Biological Sciences, Flinders University, GPO Box 2100, Adelaide, SA 5001, Australia; ^2^Marine Ecology Research Center, School of Environment, Science and Engineering, Southern Cross University, P.O. Box 157, Lismore, NSW 2480, Australia; ^3^Flinders Centre for Innovation in Cancer, Flinders University, GPO Box 2100, Adelaide, SA 5001, Australia

## Abstract

Marine molluscs from the family Muricidae are under development as a potential medicinal food for the prevention of colon cancer and treatment of gynaecological cancers. Here we report the outcome of the first *in vivo* toxicity assessment on an anticancer extract from a muricid mollusc containing brominated indole derivatives. Mice received the concentrated lipophilic extract by daily oral gavage over a two-week period. Mortality or clinical toxicity symptoms resulting from the extract were not detected during the trial, and there was no difference in the body weight of treated and control mice at the end of the trial. Histological analysis revealed some evidence for mild, idiosyncratic effects on the gastrointestinal tract and liver, including necrosis, fatty change, and inflammation in a small proportion (<40%) of mice. This is likely to result from first-pass hepatic metabolism of tyrindoxyl sulphate combined with second-pass metabolism of indoles. Overall however, oral administration of muricid extract containing brominated indoles does not result in severe clinical toxicity.

## 1. Introduction

Predatory molluscs (whelks) in the family Muricidae are well known for the production of bioactive brominated indoles, which are precursors to the ancient dye Tyrian purple [1–5]. Recently, the purified brominated indoles from Muricidae have been demonstrated to induce apoptosis and specifically inhibit female reproductive cancers, with a little effect on healthy primary granulosa cells *in vitro* [2]. Lipophilic extracts derived from these molluscs have also been shown to stimulate the acute apoptotic response to DNA damage in the distal rodent colon [6]. Stimulation of the acute apoptotic response to genotoxic carcinogens (AARGC) has recently become a promising new target for the chemoprevention of colorectal cancer (CRC) as it represents a homeostatic response to DNA-adduct formation [7]. Natural products such as cannabidiol [8], prebiotics, resistant starch [9, 10], wheat bran [7, 11, 12], and selenium [13] have all been shown to enhance the AARGC. Dose-dependent enhancement of the acute apoptotic response to a genotoxic carcinogen (AARGC) was also achieved with a crude extract from Muricidae molluscs containing brominated indoles, after daily oral administration of the muricid extract over a one-month period [6]. As muricids comprise a traditional component of African [14], European [15], Mediterranean [16], and Asian [17] diets, there is excellent potential for the development of these marine molluscs as a novel medicinal food, particularly for CRC prevention.

Colorectal cancer (CRC) accounts for 65% of new cancer cases in affluent countries and ~9.4% of cancer cases worldwide, with an annual mortality rate of ~50% [18]. Over 80% of these cases [19] arise through cumulative exposure to alkylating agents [20, 21] and heterocyclic amines [22]. These exogenous carcinogens promote cell proliferation through the formation of DNA adducts and somatic mutations, which ultimately result in cell cycle and apoptotic dysregulation [23]. In western countries, CRC incidence and return are ~10-fold greater than in countries such as India, Asia, and Africa [18], where natural chemopreventatives comprise a regular part of the diet. The chemopreventative capacity and specificity of natural products such as rhein (an anthraquinone isolated from *Cassia* species) [24], curcumin (turmeric pigment), indole-3-carbinol (cruciferous vegetables), tricin (rice beans), diallyl disulphide (garlic), soybean saponin and cardamom are substantiated by their ability to modulate signal transduction pathways and induce CRC cell cycle arrest and apoptosis [25]. 

The historical and ongoing consumption of muricid meat implies an absence of symptomatic toxicity. Nevertheless, the potential for toxicity associated with ingestion of a concentrated bioactive extract requires consideration. Drug-induced injury is common in the gastrointestinal tract and liver [26], with the latter representing a major cause of drug candidate withdrawal [27]. Although *in vivo* evidence implies tyrindoleninone and/or 6-bromoisatin selectively induces apoptosis in DNA-damaged cells [6], *in vitro* assessment suggests their mode of action and selectively depends on the cell type and the concentration of bioactive brominated compounds [1, 2, 28]. 

In addition to direct toxicity, drugs may indirectly induce toxicity through the accumulation of toxic metabolites. Effective detoxification of tyrindoleninone and 6-bromoisatin is expected, as indole and isatin metabolism is an endogenous mechanism for the detoxification of metabolites generated during tryptophan metabolism by intestinal bacteria [29]. Furthermore, the cytochrome P450 isoforms CYP2A6, CYP2E1 [30], and glutathione-*S*-transferase (GST) [31] have been implemented in the hepatic debromination of xenobiotics. Although conjugates (i.e., indoxyl sulphate and glucuronide) and minor oxidative metabolites (i.e., indoxyl, 6-hydroxyindole) are generally excreted in the urine [32, 33], enzyme substrate saturation may result in the accumulation of metabolites. One example is indoxyl sulphate, which is a uraemic toxin [34] that stimulates production of reactive oxygen species (ROS) [35]. Thus, although endogenous mechanisms are in place for the metabolism of Tyrian purple precursors, the additional metabolic load from concentrated extract administration may result in hepatotoxicity. 

To facilitate the progression of bioactive extracts from muricid molluscs into clinical trials as a chemopreventative, this investigation aims to determine if daily administration of the extract induces *in vivo* gastrointestinal and hepatotoxicity. As gastric exposure results in the oxidation of tyrindoleninone to 6-bromoisatin [6], it is uncertain what proportion of the extract escaped gastric oxidation in our previous *in vivo *trial [36]. Consequently, an extract preparation containing mostly 6-bromoisatin was administered by oral gavage in this study. The extract dosages employed correlate with those known to enhance the AARGC [6].

## 2. Experimental Section 

### 2.1. Muricid Extraction and Chemical Analysis

The Muricidae *Dicathais orbita *(Neogastropoda, Mollusca) and egg masses were collected from the rocky intertidal of the Fleurieu Peninsula, and from a population held in the Flinders University marine aquarium, South Australia. The muricid extract was prepared according to Westley et al. [6] and exposed to air prior to storage at −20°C. The secondary metabolite composition of each extract batch was determined by high-performance liquid chromatography (HPLC, Waters Alliance) coupled to a mass spectrometer (MS, Micromass Quatro micro) according to Westley and Benkendorff [37]. Compounds were identified according to retention times and characteristic major and fragment ion clusters [37]. The relative concentration of each compound was calculated from the integrated peak area in diode-array using MassLynx 4.0 software and expressed as the mean percentage (±S.D.) of the total precursor composition across both extract batches.

### 2.2. *In Vivo* Rodent Model Experimental Procedure and Sample Collection

All experimental procedures were approved by the Flinders University and Southern Adelaide Health Service Animal Welfare Committee (Project no. 632/07). After a 7d acclimation period, 8-week old female C56BL/6J mice (*N* = 28) were randomly assigned to one of four groups: (1) 0.0 mg/g control (200 *μ*L of sunflower oil, *n* = 4), (2) 0.125 mg/g muricid extract in sunflower oil (*n* = 8), (3) 0.5 mg/g extract in sunflower oil (*n* = 8), and (4) 1.0 mg/g extract in sunflower oil (*n* = 8). Mice were housed (4/cage) and maintained in a temperature- and humidity-controlled animal facility with a 12 h light-dark cycle (Flinders Medical Centre, South Australia). Muricid extract was administered daily by oral gavage, and animals were fed *ad libitum* on rodent chow and autoclaved water. Animals were weighed a day prior to the first extract dose and at regular intervals throughout the 14-day experimental period. Mice were also observed for toxicity symptoms as defined in the Common Toxicity Criteria developed by the Cancer Therapy Evaluation Program [38]. These included loss of appetite, vomiting, constipation, diarrhoea, dysphagia (inability to swallow), hematemesis (vomiting blood) and hematochezia (bloody stools). Excrement samples (combined stools and urine) were collected from animals administered oil alone (control, *n* = 4) and those receiving 1.0 mg/g muricid extract (*n* = 8) on days 7 and 14. Excrement samples were pooled according to treatment dose and analysed by LCMS for the presence of unmetabolized Tyrian purple precursors and brominated compounds, as described above.

Mice were killed on day 14 by CO_2_-induced narcosis followed by cervical dislocation. Visceral organs (colon, small intestine, liver, spleen, and kidneys) were excised immediately postmortem and weighed. Organ weights are expressed as a mean percent of total body weight. Colon, small intestine, and liver were then fixed in 10% neutral buffered formalin for histological assessment. Three animals were euthanized early in accordance with Flinders University Animal Welfare approval no. 632/07 due to overgrown teeth and handling stress associated with the oral gavage procedure. Consequently, histology was only carried out on 7 animals administered 0.5 mg/g and 6 administered 1.0 mg/g muricid extract, in addition to all 8 administered 0.125 mg/mL and the 4 controls. 

### 2.3. Gastrointestinal and Hepatotoxicity Assessment

Variables assessed by histology were indicative of antimitotic or cytotoxic drug-induced toxicity [26, 38]. Triplicate sections were examined for each organ (small intestine, colon, liver) from each mouse. Small intestine and colon sections were examined with a light microscope (Zeiss, Axio Imager, A1) and digital images acquired (Zeiss, AxioCam, ICc1). Measurements (0.01 *μ*m) were obtained using Axio Vision 8.8 software (Zeiss, 2006-09). Hepatocyte sections were examined under a BH-2 light microscope (Olympus, Japan) and measurements (0.01 *μ*m) were obtained in Adobe Photoshop version 5.5 (Adobe Systems Incorporated, 1999) in accordance with a stage micrometer.

Small intestine toxicity indicators included inflammation, ulceration, haemorrhage, villous hyper/hypoplasia and mucosal atrophy. Duodenum and ileum segments (0.5 mm) were paraffin-embedded, sectioned at 5 *μ*m and stained with haematoxylin and eosin. Triplicate sections from each mouse were examined for the presence of inflammation, ulceration, haemorrhage, and atrophy. Fourteen randomly chosen villi in each section were assessed for hyper/hypoplasia, which was quantified by determining villous height (cells/villi), cell density (cells/*μ*m), and the height of crypts of Lieberkühn (*μ*m). 

Colon toxicity indicators included mucosal inflammation, crypt abscesses, ulceration, crypt hyper/hypoplasia and dysplasia. Proximal and distal colon segments (0.5 mm) were paraffin embedded, sectioned at 5 *μ*m, and stained with haematoxylin. Whole triplicate sections from each mouse were examined for the presence of inflammation, abscesses and ulcers. Hyper/hypoplasia was quantified from 14 randomly chosen complete crypts by determining the crypt height (cells/crypt) and cell density (cells/*μ*m).

Hepatotoxicity indictors included Mallory bodies, porphyrin, porphyrin, haemosiderin, lipofuscin, peliosis (blood-filled cysts)/haemorrhage, hepatocellular hyperplasia and hypertrophy, necrosis, steatosis (fatty change), and inflammation. Liver sections were paraffin embedded, sectioned at 5 *μ*m, and stained with haematoxylin and eosin for morphological examination. Steatosis was confirmed in tissue embedded in O.C.T. (Tissue-Tek) and cryostat sectioned at 10 *μ*m and stained with Oil Red O for the demonstration of neutral lipids [39]. Triplicate liver sections from each mouse were examined for the presence of Mallory bodies, porphyrin pigment, steatosis, peliosis, and necrosis. When present, necrosis was additionally classified based on location (zone 1: periportal; zone 2: midzonal; zone 3: centrilobular) and severity (single cell, focal, submassive, or massive). Hyperplasia was determined by quantifying hepatocyte density (nuclei/mm^2^) within 10 random fields (1 mm^2^) for each mouse. Inflammatory infiltrate frequency, severity (grade 1, >5 ≤10; grade 2, >10 ≤20; grade 3, >20 ≤30; grade 4, >30 leucocytes) and location (zone 1; zone 2; zone 3), was determined within 20 random fields of view. Hypo/hypertrophy was determined by measuring the diameter of 10 hepatocytes within five random fields of view for each mouse.

### 2.4. Statistical Analysis

Statistical analyses were conducted in PASW Statistics 18^TM^ (SPSS Inc., Chicago, IL., USA, 2009) for Microsoft Windows. Analysis of variance (ANOVA, *P* < 0.05) and post Hoc (Tukey HSD) pairwise analyses were performed to determine significant differences between control animals (0.0 mg/g, *n* = 4) and those administered 0.125 mg/g (*n* = 8), 0.5 mg/g (*n* = 7), or 1.0 mg/g (*n* = 6) of muricid extract in oil. Variables subjected to ANOVA (transformations given in brackets) included colon, liver, and spleen percent weight, duodenum villous height and cell density, ileum villous height, proximal colon crypt height, cell density, distal colon crypt height and cell density (log 10), and hepatocyte density, diameter (log 10) and zone 1 liver inflammation distribution. In cases where transformation failed to meet the assumptions of normality and equal variances (Levene Statistic) for ANOVA, a nonparametric Kruskal-Wallis (*P* < 0.05) followed by Mann-Whitney U pair-wise analyses between the control and each dose were employed (Bonferroni correction *α* = 0.017). Nonparametric analyses were performed on the following variables: total weight change, small intestine and kidney percent weight, duodenum and ileum crypt of Lieberkühn depth, ileum cell density, liver sinusoid diameter, and inflammation severity (grade 1–4) and distribution (zone 2 and 3).

## 3. Results and Discussion

### 3.1. Muricid Extract and Excrement Chemical Composition

Gastrointestinal toxicity and hepatotoxicity was assessed after oral administration of an extract preparation that had been exposed to air, as gastric exposure [6] and boiling the snails for consumption (Benkendorff unpublished data), results in tyrindoleninone oxidation to 6-bromoisatin. Analysis of the extract by LCMS confirmed the dominance of 6-bromoisatin (*t*_*R*_ = 6.4 min; ES–  *m/z* 224, 226), with a mean relative concentration of 67.40 ± 12.51%, in our preparation, which is similar to that observed postgastric exposure [6]. Four other brominated indole precursors of Tyrian purple were also detected, which is consistent with previous reports on the composition of muricid extracts [1, 2, 4, 37]. Tyrindolinone (*t*_*R*_ = 9.5 min; ES−  *m/z* 302, 304; 11.32 ± 7.22%), tyriverdin (*t*_*R*_ = 12.0 min; ES−  *m/z* 511, 513, 515; 7.36 ± 3.49%), tyrindoleninone (*t*_*R*_ = 11.2 min; ES+  *m/z* 256, 258; 7.04 ± 0.18%), and tyrindoxyl sulphate (*t*_*R*_ = 5.5 min; ES−  *m/z* 336, 338; 6.88 ± 1.61%) were present in descending order of relative concentration. Analysis of extracts from excrement samples collected on day 7 or 14 from mice receiving oil alone (control), or 1.0 mg/g muricid extract, failed to reveal the presence of any HPLC peaks with brominated ions corresponding to Tyrian purple precursors in the mass spectrometer. This suggests complete metabolism of the bioactive indoles *in vivo*. 

### 3.2. *In Vivo* Rodent Model General Observations and Weight

This investigation constitutes the first *in vivo *toxicological assessment of bioactive extracts from muricid molluscs, as a novel and promising medicinal food for the chemoprevention of CRC. No visible signs of ill effects were observed for any of the mice, and the general condition of mice that received a daily dose of 0.125 mg/g, 0.5 mg/g, or 1.0 mg/g muricid extract was similar to control mice. There was no mortality or toxicity symptoms such as loss of appetite, vomiting, constipation, diarrhoea, dysphagia, hematemesis, or hematochezia [38] after daily administration of the muricid extract over a 14 d period. Animals from all groups developed an oily sheen to their coat. 

**Figure 1:**
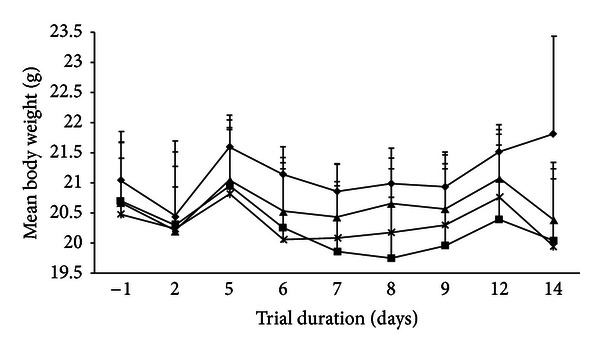
Comparison of mean (±S.D.) progressive body weight over the trial duration for control mice (◆, *N* = 4) and mice administered 0.125 mg/g (■, *N* = 8), 0.5 mg/g (▲, *N* = 7), and 1.0 mg/g (×, *N* = 6) muricid extract in sunflower oil daily.

Mean body weight decreased for all groups after day 2, before attaining a plateau (Figure 1). After day 9, the weight of control animals steadily increased, while after day 12 the weight of all treatment animals decreased to below their start weight (Figure 1). Nevertheless, no significant differences in mean total weight change were detected (*P* = 0.377). Treated mice showed some small but statistically significant changes in organ weight. Small intestine weight displayed a dose-dependent increase (Table 1), with a significant increase of 0.09 g (0.48% of body weight) detected between animals administered 0.125 mg/g and 1.0 mg/g extract (*P* = 0.008). Mean colon weight was similar between control animals and those administered 0.125 mg/g and 0.5 mg/g extract (Table 1). A significant increase of 0.03 g in colon weight (0.24% of body weight) was apparent in animals receiving 1.0 mg/g extract in comparison to animals in control (*P* = 0.034) and 0.5 mg/g extract groups (*P* = 0.034). The liver of mice administered the low extract dose of 0.125 mg/g were on average 0.25 g smaller than control mice (0.82% of body weight, *P* = 0.004) and 0.19 g smaller than mice administered the top extract dose of 1 mg/g (0.97% of body weight, *P* < 0.001) (Table 1). Mean kidney weight was significantly greater in animals administered 1.0 mg/g extract than control animals (*P* = 0.001) and those receiving 0.125 mg/g (*P* < 0.001) and 0.5 mg/g (*P* < 0.001) muricid extract, but only by 0.02 (0.22%), 0.04 (0.25%), and 0.03 g (0.22%) in kidney weight (% body weight), respectively, (Table 1). No significant differences in mean spleen weight were observed between control animals and all treatment groups (Table 1, *P* = 0.194). 

### 3.3. Gastrointestinal Effects: Small Intestine

Drug-induced gastrointestinal toxicity depends on the mode of drug absorption and the capacity and rate of first-pass intestinal metabolism. The small intestine is the predominant site of drug absorption [40] and contains various cytochrome P450 monooxygenases (CYP) (phase I) and conjugation enzymes (phase II) [41]. First-pass intestinal metabolism only occurs during transcellular absorption of lipophilic compounds, while small or hydrophilic molecules undergo paracellular absorption and first-pass hepatic metabolism [41]. As tyrindoleninone and 6-bromoisatin are lipophilic, transcellular absorption is expected, which may facilitate direct toxicity in enterocytes. 

**Figure 2:**
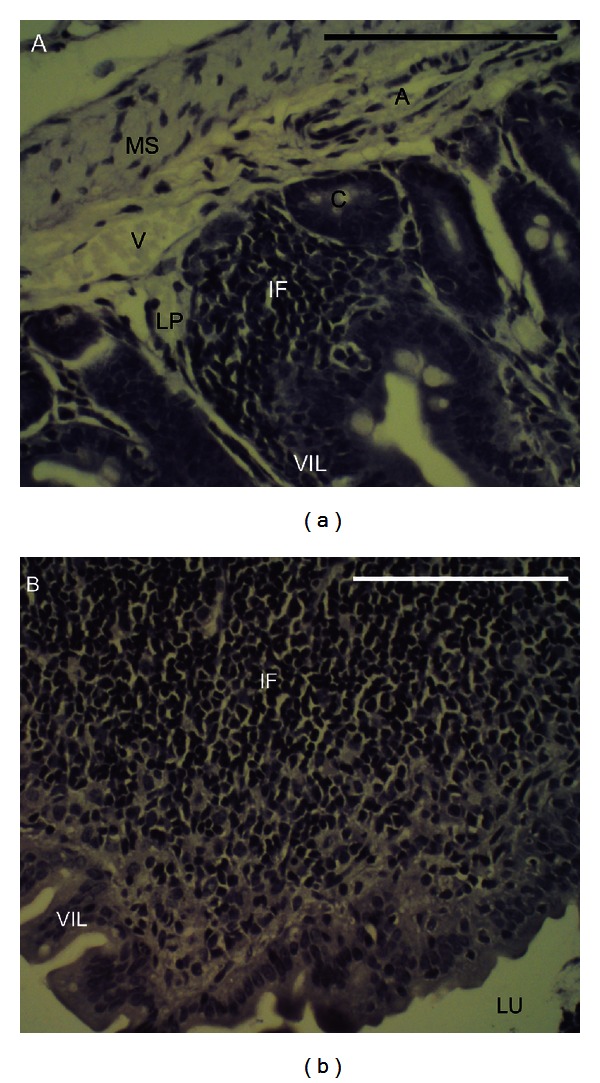
Transverse sections of small intestinal abscesses stained with haematoxylin. (a) Duodenal abscess with mild inflammation and localised crypt atrophy in a mouse administered 0.5 mg/g muricid extract, and (b) an abscess in the ileum of a mouse receiving 0.125 mg/g muricid extract characterised by chronic inflammation and extensive crypt and villous atrophy. Abbreviations: A, artery; C, crypt of Lieberkühn; IF, inflammatory infiltrate; LP, lamina propria; LU, lumen; MS, muscularis; V, vein; VIL, villi.

Some evidence for possible gastrointestinal effects associated with the muricid extract was detected in the small intestine. Histologically, gastrointestinal toxicity in the small intestine was characterised by villous hyperplasia in the duodenum. No haemorrhage or ulcers were observed in duodenum and ileum sections from control mice or any of the mice administered muricid extract. Abscesses associated with localised crypt atrophy and mild to chronic inflammation of the lamina propria were only observed in one of the seven mice administered 0.5 mg/g of the muricid extract (Figure 2(a)). The duodenum of control animals and those administered 0.125 mg/g and 1.0 mg/g muricid extract failed to display any signs of inflammation or atrophy. The mean height of villi in the duodenum of animals receiving 0.125 mg/g (*P* = 0.015), 0.5 mg/g (*P* = 0.010), and 1.0 mg/g (*P* = 0.018) extract was significantly greater than that of control animals (Table 2). Villous epithelial cell density was significantly greater in mice receiving 0.5 mg/g (*P* < 0.001), and 1.0 mg/g (*P* = 0.023) extract in comparison to control mice (Table 2). Villus cell density in the duodenum of mice treated with 0.125 mg/g extract was not significantly different to the controls (*P* > 0.05). No clear trend between duodenum crypt depth and extract dose was apparent (Table 2). However, mice administered 0.125 mg/g (*P* < 0.001) and 1.0 mg/g (*P* < 0.001) extract had significantly shortened crypts (~11 *μ*m) compared to control mice. 

No signs of inflammation or atrophy were observed in the ileum of control animals and those administered the highest dose of muricid extract (1 mg/mL). However, chronic inflammation together with crypt and villous atrophy occurred in one of eight mice administered 0.125 mg/g of muricid extract, and mild inflammation associated with crypt atrophy was observed in the ileum of 2/7 mice receiving 0.5 mg/g extract (Figure 2(a)). Focal necrosis within the basal region of villi was also evident in the ileum of one mouse receiving 0.125 mg/g extract. This response was associated with chronic inflammation of the lamina propria and widespread crypt atrophy resulting in villous flattening and a decrease in villous height (Figure 2(b)). Ileum cell height and density displayed an inconsistent response (Table 2). No significant effects were observed at the two higher doses of extracts. However in mice administered 0.125 mg/g extract, mean villous height was significantly less (*P* = 0.038) and cell density was significantly greater (*P* = 0.006) than in control animals (Table 2). Ileum crypt depth was significantly less than controls in mice administered 1.0 mg/g (*P* < 0.001).

Overall, mucosal atrophy and inflammation were absent from the small intestines of all control mice and mice administered the highest dose of 1.0 mg/g muricid extract. No hemorrhage or ulcers were observed, but a low incidence of inflammation and atrophy was detected in the duodenum and ileum of one to two mice administered lower doses of the extract. The lack of dose dependence suggests that these extract-induced gastrointestinal effects are idiosyncratic. Many anticancer drugs are known to induce mucosal inflammation, atrophy and necrosis [26]. For example, methotrexate is associated with crypt and villous atrophy as a consequence of apoptosis and S-phase cell cycle inhibition [42, 43]. Brominated isatins, including 6-bromoisatin, induce apoptosis and inhibit cell proliferation in a variety of cancer cell lines [2, 28]. A semipurified muricid extract has also been shown to reduce non-transformed intestinal epithelial cell (IEC-6) viability by >80% *in vitro* [1]. However, this *in vivo *study indicates a significant increase in enterocyte density in the duodenum at the two higher extract doses, and in the ileum at 0.5 mg/mL. A concurrent increase in villous height and cell density is usually indicative of mucosal hyperplasia [26]. Hyperplasia can arise through compensatory cell proliferation, which occurs as a regenerative response to villous atrophy [26]. Although mucosal atrophy was generally localised, it is possible that significant increases in cell density and villous height reflect regenerative proliferation. Intestinal metabolism of 6-bromoisatin may promote intestinal hyperplasia, as isatin is known to induce hypermetabolism in neuroblastoma cells [44]. Furthermore, isatin is reduced to the uraemic metabolite dioxindole by NADPH-P450 reductase (NPR) [33], which is highly expressed in enterocytes [45]. However, reduction of 6-bromoisatin to isatin in the intestine is unlikely as GST-mediated debromination is typically a phase II postoxidative reaction [31]. Thus, 6-bromoisatin is likely to avoid significant metabolism until entry to the liver. 

Transcellular absorption coupled with a limited capacity for indole metabolism in the intestinal mucosa also provides a mechanistic foundation for enterocyte toxicity induced by tyrindoleninone. Of the enzymes required for xenobiotic debromination and endogenous indole metabolism, glutathione-*S*-transferase (GST) [46], and most likely CYP2C19 occur in the intestinal mucosa [47]. However, tyrindoleninone oxidation is likely to be negligible or variable, as CYP2C19 only plays a minor role in indole oxidation [33] and is polymorphically expressed [48]. As GST-mediated debromination is a phase II process that occurs after oxidation by CYP2E1 [31], debromination capacity may also be limited. Consequently, brominated indoles within the muricid extract are predicted to undergo minimal intestinal metabolism. Previous studies have demonstrated the enhancement of the AARGC in the distal colon by the muricid extract [6], which provides further evidence for limited metabolism of the bioactive brominated indoles within the small intestine. 

### 3.4. Gastrointestinal Effects: Colon

**Figure 3:**
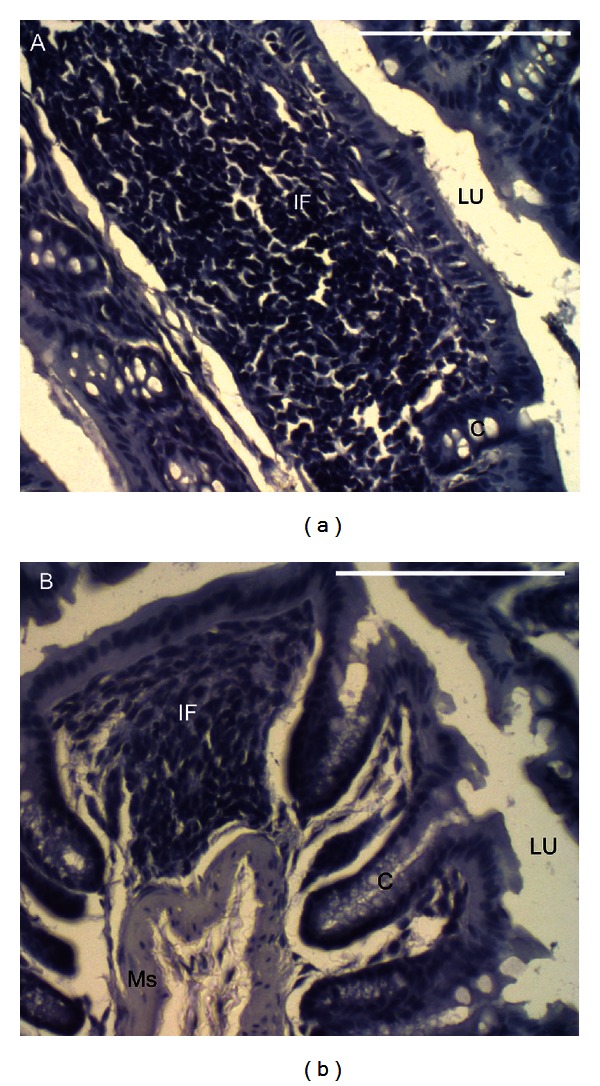
Transverse sections of colon abscesses stained with haematoxylin. (a) Proximal colon abscess in a control mouse (0.0 mg/g muricid extract) characterised by chronic inflammation and crypt atrophy, and (b) a distal colon abscess with mild inflammation and localised crypt atrophy in a mouse administered 1.0 mg/g muricid extract. Abbreviations: C, crypt; IF, inflammatory infiltrate; LU, lumen; MS, muscularis. Scale bars = 100 *μ*m.

In contrast to the small intestine, there were no histological signs of toxicity within the proximal or distal colon, such as hemorrhage, atrophy, or changes in crypt height. No evidence of haemorrhage or ulcers was revealed after examination of all 75 proximal and 75 distal colon sections in any of the control mice or mice administered muricid extract. No significant differences in proximal colon crypt height (*P* = 0.577) or cell density (*P* = 0.086) were detected between control mice and those receiving muricid extract (Table 3). Similarly, no significant differences in distal colon crypt height (*P* = 0.442) or cell density (*P* = 0.960) were detected between control mice and those receiving muricid extract (Table 3). Abscesses consisting of mild to chronic inflammation and crypt atrophy were observed in the proximal colon of one control mouse (Figure 3(a)), as well as distal colon of two control mice. Abscesses were also detected in the proximal colon of one mouse each in the 0.125 and 1.0 mg/g extract treatment groups, in three mice administered 0.5 mg/g of muricid extract, and one mild case in the distal colon for each of the 0.125 mg/g and 1.0 mg/g treatment mice (Figure 3(b)). Two out of seven mice treated with 0.5 mg/g muricid extract presented chronic inflammation in the distal colon. Although abscesses and inflammation were present in the colon of a small proportion of mice, their presence in both control and treated animals suggests that these abscesses were unlikely to be a toxic response to the muricid extract.

The observed lack of toxicity of the muricid extract in the colon is a positive sign for potential use in the prevention or treatment of colon cancer. Although the potential for idiosyncratic toxicity in the small intestine is of concern, mice in our previous *in vivo *study progressively increased in weight with no significant difference in final weight between treatment and control groups after 4 weeks of daily extract administration [6]. This suggests that any idiosyncratic effect has a negligible impact on intestinal absorption and nutritional status. Direct consumption of the muricid molluscs, or administration of the bioactive muricid extract with food, may provide a means of reducing the potential idiosyncratic gastrotoxicity, as food increases circulation and intestinal metabolism thereby decreases drug residence time [41]. 

### 3.5. Hepatotoxicity

Due to the reduced diversity and concentration of P450 enzymes in intestinal microsomes [49], intestinal metabolism of 6-bromoisatin and tyrindoleninone is predicted to be minimal. However upon entry to the liver, detoxification and conjugation in preparation for urinary excretion are expected to progress in a similar manner to that described for indole and isatin metabolites generated during tryptophan metabolism [29, 33]. The liver is the principle organ of biotransformation and contains cytochrome P450 and conjugation enzymes [26]. Consequently, hepatotoxicity is a major cause of preclinical, clinical, and approved drug withdrawal [27]. In this study, evidence for hepatotoxicity was detected in less than 40% of all mice administered the muricid extract and together with a lack of dose dependence suggests hepatic injury is idiosyncratic.

**Figure 4:**
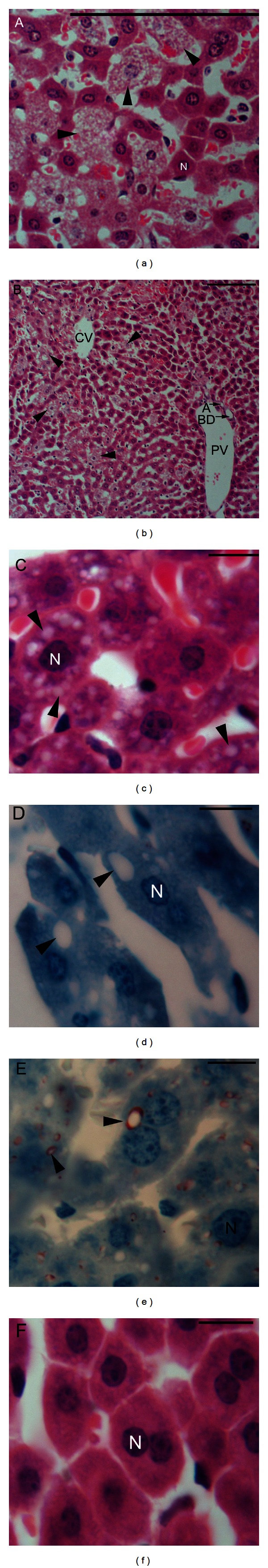
Transverse liver sections showing (a) focal necrosis (arrow head), (b) submassive necrosis (arrow head), and (c) macro- and microvesicular change (arrow head) in mice administered 0.125 mg/g muricid extract stained in haematoxylin and eosin, (d) unstained macrovesicular inclusions (arrow heads) and (e) microvesicular fatty inclusions stained with Oil Red O (arrow heads) in mice administered 0.125 mg/g muricid extract, and (f) normal hepatocytes in a control mouse stained with haematoxylin and eosin. Abbreviations: A, artery; BD, bile duct; CV, central vein (centrilobular, zone 3); N, nucleus; PV, portal vein (periportal, zone 1). Scale bars = 100 *μ*m (a-b) and 10 *μ*m (c)–(f).

No evidence for the presence of Mallory bodies, porphyrin, haemosiderin, lipofuscin, or accumulated bile was found after examination of all 75 triplicate liver sections from control mice and mice administered muricid extract. However, focal haemorrhage was evident in one of four control animals and one of eight mice administered 0.125 mg/g muricid extract. Three mice at each of the higher doses (0.5 mg/g, *n* = 7; 1.0 mg/g, *n* = 6) presented focal haemorrhage in the liver. Necrosis was observed in 2/8 and 1/6 mice administered 0.125 mg/g and at 1.0 mg/g extract, respectively. Focal centrilobular (zone 3) necrosis was present in mice administered 1.0 mg/g and 0.125 mg/g extract (Figure 4(a)). Necrosis within another mouse administered 0.125 mg/g extract was submassive in nature, with a centrilobular distribution extending to adjacent periportal (zone 1) regions (Figure 4(b)). Macrovesicular and microvesicular cytoplasmic changes (Figure 4(c)) were observed within hepatocytes proximal to Glisson's capsule in some of the mice administered the lower doses of muricid extract. Macrovesicular changes occurred in half the mice administered 0.125 mg/g (*n* = 8) and 2/7 mice administered 0.5 mg/g muricid extract. Although these inclusions failed to stain postfixation with osmium tetroxide for unsaturated lipids (Figure 4(d)), peripheral staining with Oil Red O for neutral lipids was observed on occasion (Figure 4(e)). Microvesicular changes stained consistently with Oil Red O (Figure 4(e)) and were evident in three and two mice administered 0.125 mg/g and 0.5 mg/g muricid extract, respectively.

**Figure 5:**
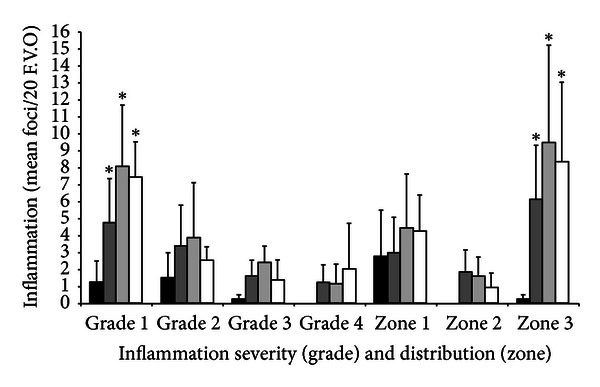
Comparison of the severity and distribution of hepatic inflammatory infiltrates in control mice (black bars, *N* = 4) and mice administered 0.125 mg/g (dark grey filled bars, *N* = 8), 0.5 mg/g (light grey filled bars, *N* = 7), and 1.0 mg/g (white bars, *N* = 6) muricid extract on a daily basis. Values are the mean frequency of foci/20 fields of view at 200x magnification. Inflammation severity is graded as follows grade 1 (>5 ≤10); grade 2 (>10 ≤20); grade 3 (>20 ≤30), grade 4 (>30) lymphocytes/foci. The distribution of foci is categorised by acinous zone: zone 1, periportal (portal triad); zone 2, periportal centrilobular; zone 3, centrilobular (central vein).

Macrovesicular change coincided with microvesicular change in all cases. Necrosis and fatty change were not observed in control animals (Figure 4(f)) or any of the mice administered the high dose of 1.0 mg/g extract. Mean frequency of inflammatory foci was significantly greater in mice administered muricid extract relative to the controls (Table 4, *P* < 0.001). Similarly, the mean frequency of grade 1, 2, and 3 foci increased in all doses of muricid extract, relative to the controls (Figure 5). Grade 4 inflammatory foci were only observed in treatment groups and were of greatest frequency in animals administered 1.0 mg/g extract (Figure 5). Despite these trends, only the frequency of grade 1 foci was significantly greater in mice administered extract than in control animals (*P* = 0.001). The frequency of zone 3 (centrilobular) inflammatory foci also significantly increased in mice administered muricid extract compared to control animals (*P* = 0.001). The frequency of zone 1 (periportal) and zone 2 (midzonal) inflammatory foci displayed no significant differences with extract administration relative to controls (Figure 5).

Hepatocyte density displayed an inconsistent dose response in mice treated with muricid extract (Table 4). Hepatocyte density peaked in mice administered the lowest extract dose of 0.125 mg/g and was significantly greater than in control mice at this dose and at 0.5 mg/g (*P* ≤ 0.001). In contrast, hepatocyte density was similar to controls in mice administered the higher dose of 1 mg/g extract. Hepatocyte diameter was the lowest in mice treated with 0.125 mg/g extract (Table 4), with a significantly larger hepatocytes in control mice in comparison to all mice treated with muricid extract (*P* < 0.001 for all doses). Sinusoid diameter was significantly greater in mice administered the extract (*P* < 0.001) than in control animals. 

Microvesicular and macrovesicular steatosis, necrosis and inflammation are hallmarks of nonalcoholic steatohepatitis (NASH) [50]. Steatosis, as confirmed by Oil Red O, was observed in a few mice administered the two lower doses of muricid extract but was not observed at all in the high-dose treatment group. Necrosis was only observed in two mice at the lowest dose whereas focal hemorrhages were more frequent at the two higher doses. Inflammation and a significant increase in the frequency of centrilobular inflammatory foci were also evident in some mice administered the extract. As these lesions were absent from control mice, it appears that daily administration of muricid extract does have the potential to induce idiosyncratic hepatic injury. Absence of accumulated bile, Mallory bodies, porphyrin and haemosiderin suggests that bile duct obstruction is not responsible for muricid extract-induced hepatotoxicity.

Indoxyl sulphate is predicted to be a significant product of muricid extract metabolism and could be responsible for some of the observed hepatotoxicity. Indoxyl sulphate is an endogenous conjugation product of tryptophan metabolism and is excreted in the urine [29]. Accumulation of indoxyl sulphate is known to stimulate production of reactive oxygen species (ROS) [35], which is strongly implicated in the development of nonalcoholic fatty liver disease (NAFLD) and progression to NASH, cirrhosis and cancer [51, 52]. Tyrindoxyl sulphate is a minor constituent of the muricid extract and is predicted to undergo paracellular absorption and hepatic debromination to indoxyl sulphate by CYP2A6, CYP2E1 [30] or GST [31]. Debromination, oxidation [53] and conjugation [29] of other muricid indoles (>25% of extract) may also yield indoxyl sulphate. Accumulation of indoxyl sulphate may be further enhanced by its binding affinity to serum albumin and the influence of indomethacin, a nonsteroidal antiinflammatory indole which inhibits renal uptake [34]. Methanethiol generation during hepatic metabolism of tyrindoleninone, tyrindolinone, or tyriverdin may also contribute to steatosis. Metabolism of methionine yields toxic methanethiol, which inhibits enzymes that provide protection from peroxidative damage [54]. As excessive dietary intake increases lipid peroxidation in the liver [55], it is possible that methanethiol derived from the muricid extract contributes to lipid accumulation in some mice. Although muricid extract may result hepatic injury synonymous with NASH, observations of hepatocyte hyperplasia in mice administered the extract suggest a regenerative response to hepatic injury [26].

In addition to hepatic injury, indoxyl sulphate accumulation may also result in renal injury, Indoxyl sulphate induces glomerular hypertrophy, stimulates cell proliferation [35] and inhibits active tubular secretion [56]. This is partially supported by the significant increase in kidney weight observed in mice administered 1.0 mg/g of the muricid extract (Table 1). The potential for hepatic injury and impaired renal function is of concern to the development of crude muricid extract as an oral chemopreventative. Quantification of indoxyl sulphate after extract administration is required to determine the potential for accumulation to toxic levels. Further hydrolysis and purification of the muricid extract to reduce the amount of tyrindoxyl sulfate and indoles in favor of 6-bromoisatin may help reduce the potential for liver and kidney damage. 

Interindividual variability and a lack of consistent dose-dependent responses to the muricid extract imply idiosyncrasy. Idiosyncratic hepatotoxicity typically occurs as a result of individual immunologic or metabolic susceptibility [57]. As all mice administered the muricid extract appeared consistent in health, with no signs of rash or fever that would imply an immune reaction, idiosyncratic hepatotoxicity is likely to be of metabolic origin. The P450 isoforms CYP2E1 [53] and CYP2C19 [48] exhibit significant interindividual variation and are known to be involved in endogenous indole metabolism and xenobiotic debromination. As indole oxidation is catalysed by CYP2E1 [53], and to a lesser extent CYP2C19 [33], variability in the expression of these isoforms may alter the kinetics of metabolite detoxification. Furthermore, nutritional status of individual mice could influence metabolite absorption, bioavailability, and metabolism. Gastric emptying into the intestinal lumen stimulates the production of bile acids and pancreatic enzymes, which can act as wetting agents, enhance fat solubility, and alter cell membrane functions [41]. Intestinal circulation is also increased in a fed state, which increases the rate of drug metabolism and decreases intestinal residence time [41]. As mice were fed *ad libitum*, intestinal metabolism of the bioactive compounds and hence the incidence and severity of extract-induced gastrointestinal effects will depend on the timing of individual food intake, in relation to extract administration. 

## 4. Conclusions 

The findings of this investigation indicate that daily consumption of muricid extract has no major visible signs of toxicity but can have mild gastrointestinal effects and induce idiosyncratic hepatotoxicity in some mice. The lack of co-occurrence in individuals suggests that observations of gastrointestinal toxicity and hepatotoxicity are unrelated. Gastrointestinal effects were characterised by heavier small intestines and villous hyperplasia in the duodenum. Extract-induced hepatotoxicity was primarily characterised by idiosyncratic injury including, steatosis, necrosis, and inflammation. As drug-induced hepatotoxicity is a major cause of preclinical drug withdrawal [27], research into potential protective agents and the origin of idiosyncratic expression is paramount to the development of muricid extract as a CRC chemopreventative. It is worth noting that muricid molluscs are regularly consumed in many countries, with no previous reports of toxicity. Consequently, the observed toxic effects may be less likely to occur when the bioactive constituents are ingested in lower concentrations in association with muricid meat. Therefore, future development of the Muricidae as a medicinal food for cancer prevention will require further research on the purified active constituents and alternative delivery regimes, such as incorporation of the extract into a controlled diet rather than oral gavage. 

## Figures and Tables

**Table 1:** Mean (±S.D.) organ weight as a percent of total body weight in control mice (0.0 mg/g, *N* = 4) and mice administered 0.125 mg/g (*N* = 8), 0.5 mg/g (*N* = 7) and 1.0 mg/g (*N* = 6) of muricid extract (ME). Significant differences (*P* < 0.05) between control and ME-treated mice are indicated by *.

ME dose (mg/g)	Percent of body weight (%)				
	Colon	Small intestine	Liver	Kidney	Spleen
0.000	0.68 ± 0.22	3.47 ± 1.33	5.11 ± 0.66	1.43 ± 0.08	0.36 ± 0.03
0.125	0.78 ± 0.07	4.17 ± 0.29	4.29 ± 0.16*	1.40 ± 0.08	0.41 ± 0.04
0.500	0.71 ± 0.13	4.53 ± 0.74	4.76 ± 0.33	1.43 ± 0.09	0.39 ± 0.05
1.000	0.92 ± 0.09*	4.65 ± 0.19*	5.26 ± 0.26	1.65 ± 0.04*	0.42 ± 0.04

**Table 2:** Mean (±S.D.) morphometric values for small intestine toxicity indicators in control mice (0.000 mg/g, *N* = 4) and mice administered 0.125 mg/g (*N=8*), 0.5 mg/g (*N=7*), and 1.0 mg/g (*N=6*) muricid extract (ME). Significant differences between controls and extract doses are indicated by * (Tukey, *P* < 0.05; Mann-Whitney, *P* < 0.017).

Intestine region	ME dose (mg/g)	Villous height (cells/villi)	Villous cell density (cells/*µ*m)	Crypt depth (*µ*m)
Duodenum	0.000	74.25 ± 15.43	0.18 ± 0.06	75.81 ± 16.36
	0.125	82.29 ± 16.32*	0.19 ± 0.07	65.18 ± 10.69*
	0.500	82.85 ± 18.88*	0.24 ± 0.07*	74.12 ± 14.79
	1.000	82.58 ± 13.73*	0.21 ± 0.05*	64.62 ± 14.45*

Ileum	0.000	38.96 ± 8.70	0.19 ± 0.06	62.33 ± 14.08
	0.125	35.51 ± 8.23*	0.22 ± 0.11*	60.68 ± 9.81
	0.500	37.68 ± 7.39	0.21 ± 0.06	64.13 ± 15.83
	1.000	37.07 ± 6.78	0.18 ± 0.03	70.19 ± 17.27*

**Table 3:** Mean (±S.D.) morphometric values for colon toxicity indicators in control mice (0.000 mg/g extract, *N=4*) and mice administered 0.125 mg/g (*N=8*), 0.5 mg/g (*N=7*), and 1.0 mg/g (*N=6*) of muricid extract (ME).

Colon region	ME dose (mg/g)	Crypt height (cells/crypt)	Crypt cell density (cells/*µ*m)
Proximal	0.000	13.66 ± 1.90	0.18 ± 0.05
	0.125	13.78 ± 2.31	0.17 ± 0.05
	0.500	14.00 ± 2.64	0.19 ± 0.06
	1.000	13.50 ± 2.71	0.18 ± 0.05

Distal	0.000	22.77 ± 3.34	0.21 ± 0.07
	0.125	23.23 ± 3.53	0.19 ± 0.05
	0.500	22.43 ± 3.81	0.18 ± 0.04
	1.000	23.05 ± 4.01	0.19 ± 0.04

**Table 4:** Frequency and mean morphometric values (±S.D.) for hepatotoxicity indicators in control mice (0.0 mg/g, *N=4*) and mice administered 0.125 mg/g (*N=8*), 0.5 mg/g (*N=7*), and 1.0 mg/g (*N=6*) of muricid extract (ME). Significant differences between control and treated mice are indicated by * (Tukey, *P* < 0.05; Mann-Whitney, *P* < 0.017. Abbreviations: FOV: field of view; IF: inflammatory foci.

ME dose (mg/g)	Hepatocyte density (cells/mm^2^)	Hepatocyte diameter (*µ*m)	Sinusoid diameter (*µ*m)	IF frequency (foci/20 FOV)
0.000	23.73 ± 4.31	18.98 ± 3.20	4.47 ± 1.07	0.15 ± 0.42
0.125	30.16 ± 5.51*	15.16 ± 2.43*	5.93 ± 2.09*	0.55 ± 0.90*
0.500	27.68 ± 5.95*	16.46 ± 2.65*	5.91 ± 1.57*	0.73 ± 1.11*
1.000	24.80 ± 4.98	17.29 ± 2.88*	5.43 ± 1.36*	0.76 ± 0.07*
